# Concentration and purification of enterovirus 71 using a weak anion-exchange monolithic column

**DOI:** 10.1186/1743-422X-11-99

**Published:** 2014-05-27

**Authors:** Ashok Raj Kattur Venkatachalam, Milene Szyporta, Tanja Kristin Kiener, Premanand Balraj, Jimmy Kwang

**Affiliations:** 1 Animal Health Biotechnology, Temasek Lifesciences Laboratory, National University of Singapore, Singapore 117604, Singapore; 2 Department of Microbiology, Yong Loo Lin School of Medicine, National University of Singapore, Singapore 117545, Singapore

**Keywords:** Enterovirus 71, PEG precipitation, DEAE monolithic column

## Abstract

**Background:**

Enterovirus 71 (EV-71) is a neurotropic virus causing Hand, Foot and Mouth Disease (HFMD) in infants and children under the age of five. It is a major concern for public health issues across Asia-Pacific region. The most effective way to control the disease caused by EV-71 is by vaccination thus a novel vaccine is urgently needed. Inactivated EV-71 induces a strong, virus-neutralizing antibody response in animal models, protecting them against a lethal EV-71 challenge and it has been shown to elicit cross-neutralizing antibodies in human trials. Hence, the large-scale production of purified EV-71 is required for vaccine development, diagnosis and clinical trials.

**Methods:**

CIM^®^ Monolith columns are single-piece columns made up of poly(glycidyl methacrylate *co*-ethylene dimethacrylate) as support matrix. They are designed as porous channels rather than beads with different chemistries for different requirements. As monolithic columns have a high binding capacity, flow rate and resolution, a CIM^®^ DEAE-8f tube monolithic column was selected for purification in this study. The EV-71 infected Rhabdomyosarcoma (RD) cell supernatant was concentrated using 8% PEG 8000 in the presence of 400 mM sodium chloride. The concentrated virus was purified by weak anion exchange column using 50 mM HEPES + 1 M sodium chloride as elution buffer.

**Results:**

Highly pure viral particles were obtained at a concentration of 350 mM sodium chloride as confirmed by SDS-PAGE and electron microscopy. Presence of viral proteins VP1, VP2 and VP3 was validated by western blotting. The overall process achieved a recovery of 55%.

**Conclusions:**

EV-71 viral particles of up to 95% purity can be recovered by a single step ion-exchange chromatography using CIM-DEAE monolithic columns and 1 M sodium chloride as elution buffer. Moreover, this method is scalable to purify several litres of virus-containing supernatant, using industrial monolithic columns with a capacity of up to 8 L such as CIM^®^ cGMP tube monolithic columns.

## Background

Enterovirus 71, a close relative of polioviruses, was first isolated in California, USA in 1969 [1]. Since then it has become a major public health issue across Asia-Pacific region causing Hand, Foot and Mouth Disease (HFMD) in infants and children under the age of five [2]. It is an important neurotropic virus in Asia for which no effective vaccine is available [3]. The most effective way to control the disease caused by EV-71 is by vaccination and thus arises the need for the development of new vaccines [4]. As inactivated polio vaccine elicits long term protection against the virus, this strategy might be efficacious for chemically inactivated EV-71 as a vaccine candidate [5]. In recent years, several researchers [4,6,7] have shown that inactivated EV-71 (heat or formalin inactivation) induces a strong, viral-neutralizing antibody response in animal models, thus protecting them against a lethal EV-71 challenge.

Viruses possess various distinct characteristics some of which are: the number and distribution of positive or negative charges, distribution of aliphatic and aromatic hydrophobic residues and finally, their size. These virus characteristics can be utilized to fractionate them from other molecules [8]. The initial step in any purification process is to concentrate the molecules of interest. Precipitation by polyethylene glycol (PEG) is a widely employed method to concentrate larger proteins during the initial step of the purification process [9]. PEG, even at higher concentrations, does not interact with proteins or denature them and there is no need to remove it from the sample. PEG, due to its non-ionic nature, does not bind to ion-exchange columns and is therefore removed in the flow-through [10]. Magar and Lecomte [11] compared the use of ultrafiltration (UF) and PEG for the concentration of Bovine Diarrheal virus, where they found PEG to be superior to UF as it retains almost 100% infectivity with lower protein content. The combination of PEG precipitation and monolithic chromatography was also used for the purification of mycobacteriophage D29 [12].

Ion-exchange chromatography is widely used as an initial chromatographic procedure in which 80% of the impurities are removed and is usually followed by a polishing step. The disadvantages of bead-based media is their smaller pore size distribution (60–100 nm), where many viral particles cannot enter the matrix. This in turn affects the total binding capacity of the column. Monoliths are ready to use columns, made from porous materials, with interconnected channels forming rigid chromatographic support. They provide large adsorbing surfaces, leading to increased binding capacities while higher flow rates are achieved through the movement of particles by convective flow. These monolithic columns have the additional advantage of short separation time and flow-independent separation [13]. Moreover, the biological activity of viruses is retained [14]. Thus, the use of methacrylate based monoliths for protein purification appears to be a better alternative to conventional gradient centrifugation techniques [15-17].

Chromatographic columns used for the purification of viruses should be sanitized immediately to prevent cross-contamination of products or transmission of viruses. Sodium hydroxide is widely used for regeneration and sanitation of chromatographic columns [18]. Thus monolithic columns are stable at high alkali conditions making them useful for sanitation by sodium hydroxide. A study on the chemical and chromatographic stability of methacrylate-based monoliths (QA and DEAE) in the presence of sodium hydroxide and ethanol found that the degradation of DEAE groups is relatively small compared to QA groups even after 50 cycles of CIP procedure [19]. Because of their superior mass transfer and open porous structure, the monolith columns are able to provide very fast biospecific pair formation involving viruses that reduce the risk of product degradation [20].

The need for the production of large quantities of purified EV-71 to be used as vaccine candidates and for clinical trials drove us to find an alternative way for the purification of EV-71 with high yield and faster processing time.

## Results

### Sample preparation and concentration of virus

**Figure 1:**
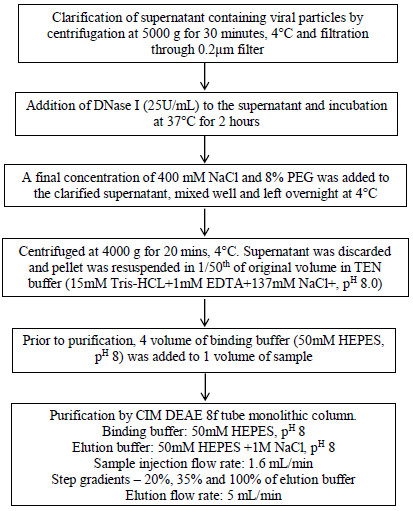
Overall process for concentration and purification of EV-71.

One litre of cell culture supernatant containing EV-71 virus collected from T175 cm^2^ tissue culture flasks was used for purification. The overall process is depicted in Figure 1. Prior to concentration, DNase was added to the supernatant to reduce viscosity [21]. Concentration by PEG 8000 yielded a mixture of large amounts of viruses and other contaminant proteins from the Dulbecco’s Modified Eagle Medium (DMEM) and RD cells used for growing the virus. The presence of more concentrated virus was evident by measuring the TCID_50_.

### Virus purification

**Figure 2:**
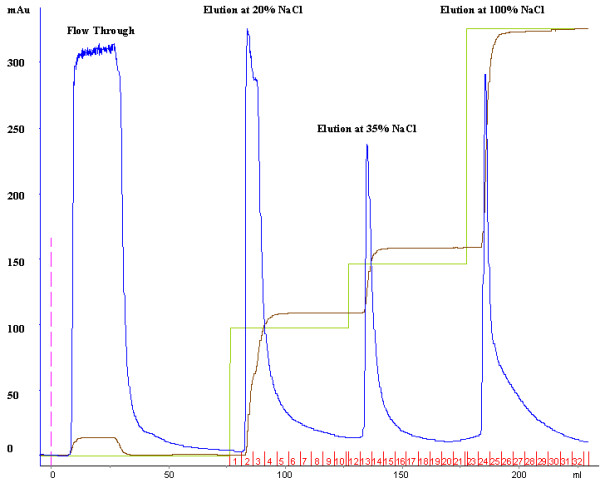
**Step gradient elution profile of EV-71 in CIM^®^ DEAE 8f tube monolith column.** Loosely bound proteins were eluted in the first peak using 20% NaCl. Pure viral particles were eluted in second peak using 35% NaCl. The last peak eluted using 100% NaCl completely removed all the tightly bound proteins from the column.

The initial experiments with linear gradient elution indicated that most of the viral particles were eluted at a salt concentration around 350 mM. Hence, subsequent experiments were done by step gradient elution using salt concentrations of 200 mM, 350 mM, and 1 M NaCl (Figure 2). In first step of the gradient process, 200 mM NaCl removed most of the contaminant proteins. This was followed by 350 mM NaCl step, where pure viral particles were eluted. Strongly bound proteins were removed during the final step using 1 M NaCl. The volume collected from the 350 mM NaCl step was approximately 30 mL, which was further concentrated using a centrifugal filtration device, followed by dialysis in NTE buffer to 4 mL.

### Determination of TCID_50_

TCID_50_ of the supernatant, PEG concentrated and purified samples was determined by an end-point dilution assay [22] on Vero cells (Table 1). The clarified culture supernatant showed a TCID_50_ of 1.27 × 10^6^ while the TCID_50_ after PEG concentration was 2.54 × 10^7^ with 80% recovery. The final recovery of the purified sample was about 55% with a TCID_50_ of 6.9 × 10^8^.

**Table 1:** Recovery of EV-71 during purification process

**Sample**	**TCID**_**50**_	**Volume (mL)**	**% final recovery**
**Supernatant**	1.27 × 10^6^	1000	100
**PEG concentration**	2.54 × 10^7^	40	80
**Purified sample**	6.9 × 10^8^	4	55

### SDS-PAGE and Western blotting

**Figure 3:**
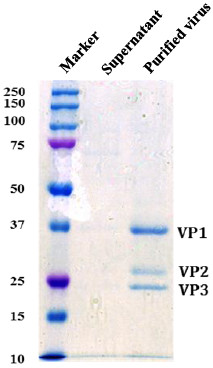
**SDS-PAGE of purified EV-71 in 10% acrylamide gel stained with Coomasive Brilliant Blue R-250 staining.** Lane 1: Bio-Rad Broad range dual colour marker. Lane 2: Unpurified culture supernatant. Lane 3: Purified EV-71. Lane 2 and 3 were loaded equally with 50 μL of sample using 6x loading dye.

**Figure 4:**
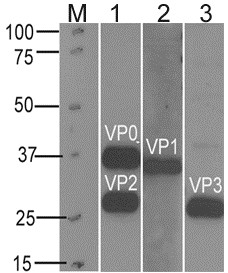
**Western blotting of purified EV-71.** M: Marker. Lane 1: anti-VP2 mouse monoclonal antibody (7C7). Lane 2: anti-VP1 mouse monoclonal antibody (mAb 51). Lane 3: anti-VP3 mouse polyclonal antibody.

Coomassie Brilliant Blue staining analysis of an SDS-PAGE gel (Figure 3) of the purified sample showed a band with the approximate molecular weight of 36 kDa and two bands between 28 and 25 kDa, which correspond to VP1, VP2 and VP3 respectively. Western blotting of purified EV-71 with anti-VP1 mouse monoclonal antibody [23], anti-VP2 mouse monoclonal antibody [24] and anti-VP3 mouse polyclonal antibody confirmed the presence of VP1, VP2 and VP3 capsid proteins in the purified sample (Figure 4). Western blotting results using the anti-VP2 monoclonal antibody showed 2 bands representing VP0 and VP2.

### Transmission electron microscopy

**Figure 5:**
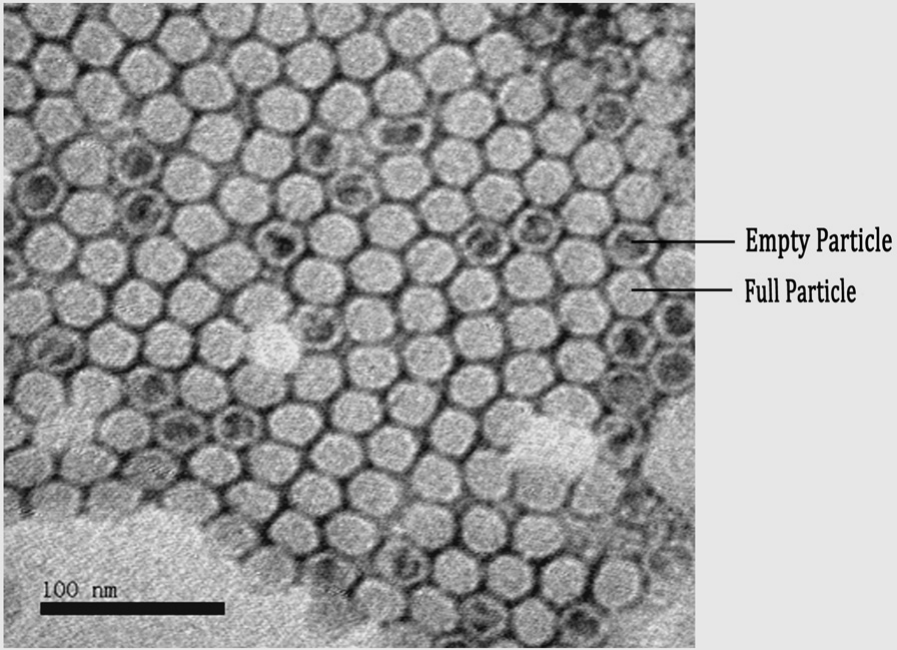
Transmission electron microscopy of purified EV-71 negatively stained with 2% phospho-tungstic acid.

The TEM analysis of the purified EV-71 particles (Figure 5) showed two kinds of viral particles, presumably full/infectious particles along with some empty particles without viral RNA [25]. The full particles were around 30 nm in size.

## Discussion

The onset and spread of EV-71 is rapid among infants and the strain varies according to the geographical location and season [26]. After a quick diagnosis of the strain type, large amounts of vaccine should be produced for use in prophylactic measures. The particular use of inactivated EV-71 particles as a potent vaccine requires high quantities of the virus to be produced, inactivated and purified as rapidly as possible. A literature survey [3,27] showed that EV-71 is preferably purified by conventional techniques such as ultracentrifugation or precipitation followed by sucrose gradient/caesium chloride centrifugation or size-exclusion chromatography, which are laborious and time consuming processes.

A serum-free cell-based EV-71 vaccine candidate was proposed by Chou et al. [3], in which a combination of sucrose-gradient ultracentrifugation and/or gel-filtration liquid chromatography purifications was studied. They employed a pilot liquid chromatography system using Sepharose Fast Flow 6 gel. Even though the procedure yielded infectious viral particles of high purity, the overall yield was only 7–10% as determined by a VP2-based quantitative ELISA. They further demonstrated that the purified, formalin-inactivated vaccine candidate was stable and could induce strong virus-neutralizing antibody responses in mice, rats, rabbits, and non-human primates. Production of bulk EV-71 vaccines in roller bottles/bioreactor using a serum-free media and its purification by Sepharose Fast Flow 6 gel was reported by Liu et al. [28] where the average recovery was about 50%, as is the case with our results with a slight (5%) increase in recovery. A 50% recovery of EV-71 during the purification by gel-filtration chromatography was also reported by Chang et al. [5] but they suggested that the chromatographic process needs to be improved due to the inconsistent results obtained.

Thus, for the bulk production of different strains of EV-71 as vaccine candidates, a number of factors should be considered. This includes the formulation of a growth medium that supports the production of a high virus titre combined with minimal downstream steps, two factors that are critical for economic viability. During infection of RD cells, we used serum-free DMEM to minimize binding of serum proteins during the purification process.

Ion-exchange columns are extensively used in downstream applications due to their efficiency and cost-effectiveness. Anion exchange columns are widely used as the first step in the purification of viruses and viral vectors because of their enhanced binding of viruses instead of contaminating proteins [21]. This might be the reason that the majority of the contaminating proteins are easily eluted in lower salt concentrations in the earlier steps of purification.

Monoliths are highly interconnected porous structures, made up of different chemistries, currently being used for purification of large biomolecules such as viruses and bacteriophages. They are considered advantageous over other columns as they have properties unaffected by flow, shorter separation times and a high binding capacity [13]. Moreover, the purification protocol is simple and the total time taken can be shortened from several days to a few hours without compromising purity. The method developed from a disk/tube monolithic column can be easily scaled-up to litres for bulk purification of biomolecules [29]. Recently, CIM DEAE monolithic columns have been used for the purification of many viruses and bacteriophages including Tomato mosaic virus [16,30], filamentous potato virus [17], Pseudomonas phage LUZ19 [31], Rubella virus [32], Staphylococcus phage ISP [33], viral vectors such as Canine Adenoviral Vectors [14], and Lentiviral Vectors [34].

Presence of three bands (VP1, VP2 and VP3) suggested of viral particles and similar results were presented by Liu et al. [27] during the production and purification of EV-71 viral particles in a serum free bioreactor system. Western blotting with anti-VP2 monoclonal antibody showed two bands (VP0 and VP2), as reported by Liu et al. using the commercial monoclonal antibody mAb 979 [35]. Our method of purification yielded some empty particles when analysed by TEM (Figure 5). The antigenicity of the empty particles may be different from native virus but they only represent a minority of the particles. Hence their presence is unlikely to have a major effect on the overall vaccine efficacy of the purified virus.

## Conclusion

Our present work demonstrated, for the first time, the purification of EV-71 particles by monolithic weak anion exchange (DEAE) chromatography where the recovery was around 55% with a 100-fold increase in virus titre and 95% purity. Further improvements in the process of EV-71 purification can be made by employing monolithic columns of different chemistries along with different buffers of varying pH. This ensures the determination of best column and buffer combination for the highest possible recovery of virus particles. Moreover, the host cell protein and DNA content of the harvest, intermediary and final product should be assessed, which is a prerequisite for use as a vaccine candidate.

## Materials and methods

### Growth and harvesting of virus

EV-71 B2 strain (7423/MS/87, GenBank # U22522.1) was used for the purification process. RD cells were grown to 80% confluency in T175 cm^2^ polystyrene tissue culture flasks (BD Falcon, USA) using Dulbecco’s Modified Eagle medium (DMEM) (Gibco, USA) supplemented with 10% foetal bovine serum (FBS) (Biowest SAS, France), 3.7 g/L sodium bicarbonate (Sigma-Aldrich, USA) and Gibco^®^ Antibiotic-Antimycotic solution. The flasks were incubated at 37°C in a humidified incubator saturated with 5% carbon dioxide. During infection of RD cells, the medium was depleted of serum (DMEM without FBS), as serum proteins might interfere with the purification steps. 30 μL of the virus sample, with a TCID_50_ of 10^6^, was used to infect RD cells in T175 cm^2^ flask containing 30 mL of serum-free DMEM which were incubated at 37°C in a humidified incubator saturated with 5% carbon dioxide.

72 hours post-infection the content of the flasks were collected, freeze-thawed 3 times and pooled to yield 1.5 litre of virus containing supernatant. The sample was then clarified by centrifugation at 5000 g for 30 minutes. The clarified supernatant was treated with DNase I (Roche, Germany), at a final concentration of 50 U/mL, and incubated at 37°C for 3 hours. Finally the sample was passed through a 0.2 μm polyether sulfone (PES) filtration unit (Nalgene, USA).The sample was then kept either at 4°C for short term or at -80°C for long term storage.

### Concentration of virus

A 1:1 (wt:vol) PEG 8000 (Merck, Germany) was prepared by adding 100 g of solid PEG to 100 mL of sterile distilled water, mixed to dissolve completely and stored at 4°C. Sodium chloride (Merck, Germany) and PEG 8000 was added to 1 litre of DNase treated virus supernatant to a final concentration of 400 mM and 8% respectively. This mixture was gently mixed using a magnetic stirrer and left overnight at 4°C. It was then filled in a 50 mL falcon tube and centrifuged at 4000 g for 30 minutes. The supernatant was discarded and 1 mL of NTE buffer (137 mM NaCl + 15 mM Tris–HCl + 1 mM EDTA, p^H^ 8.0) was added to the pellet which was allowed to stand overnight at 4°C followed by gentle resuspension using a micro pipette.

### Instrumentation and column

The purification steps were done on ÄKTApurifier 10 (GE Healthcare Life Sciences, Sweden) using an 8 mL CIM^®^ DEAE-8f tube monolithic column (BIA separations, Austria).

### Virus purification

Before commencing the purification procedure, the sample, buffers and column were brought to room temperature. Four parts of binding buffer (50 mM HEPES, p^H^ 8) was added to one part of sample and filtered through a 0.2 μm syringe filter. All the steps, prior to sample loading, were done for 5 column volume (CV) at a flow rate of 2.5 mL/min. The monolithic column was washed and equilibrated in the following order: Deionized water – 1 M NaOH – deionized water – elution buffer (50 mM HEPES + 1 M NaCl, p^H^ 8) – binding buffer (50 mM HEPES, p^H^ 8).

Prior to purification, 1 volume of the PEG concentrated sample was diluted with 4 volumes of binding buffer. To determine the salt concentration required to elute the virus particles, 2 mL of the sample was injected in to the column at a flow rate of 1.6 mL/min and a linear gradient elution was performed at a flow rate of 5 mL/min, using elution buffer. Once the appropriate salt concentration was determined, subsequent purifications were done in a step gradient mode. Briefly, 40 mL of the sample was loaded to the column at a flow rate of 1.6 mL/min. Unbound sample was washed by passing 5 CV of binding buffer. The sample was then eluted using different concentration of elution buffer as follows: 20% (200 mM NaCl), 35% (350 mM NaCl) and 100% (1 M NaCl) at a flow rate of 5 mL/min for 6.25 CV (50 mL) each. The virus sample, eluted in the second step (350 mM NaCl), as 5 mL fractions was pooled, concentrated and buffer exchanged with NTE buffer using Vivaspin 20, MWCO 100 kDa (Sartorius AG, Germany). The purified samples were stored at -80°C as 500 μL aliquots.

### Determination of TCID_50_ by immunofluorescence assay

To determine the tissue culture infective dose (TCID_50_) of the purified and unpurified samples, a 50% end-point dilution assay [22] was performed on Vero cells. Briefly, 96-well micro titre plates were seeded with Vero African green monkey kidney cells and infected with the different dilutions (10^1^ to 10^8^) of the unpurified and purified virus samples for approximately 48 h at 37°C. From the onset of cytopathic effect (CPE), cells were fixed with 4% paraformaldehyde (pH 7.4) for 20 min at room temperature. The cells were then permeabilized with 0.1% Triton in PBS for 5 min, followed by blocking in 5% milk (Skim milk powder + PBST) for 1 h. The cells were then washed with PBST and incubated with monoclonal antibody mAb 51 [23] specific to VP1 of EV-71 for 1 h at 37°C. After washing, FITC-conjugated rabbit anti-mouse (Dako, Denmark) secondary antibody was added and incubated for 1 h at 37°C. Cells were washed with PBST twice for 5 min in between the steps. Results were documented with an inverted microscope (Olympus) with Nikon ACT-1 software.

### SDS-PAGE and Western blotting

10% acrylamide gels were run at 100 volts for 100 minutes to resolve the purified samples along with a protein molecular weight marker (Bio-Rad, USA) and used for coomasive brilliant blue R-250 staining. Resolved proteins in SDS-PAGE gels were transferred to a nitrocellulose membrane and non-specific sites were blocked with 5% milk. In-house monoclonal antibodies against VP1 (mAb 51) [23], VP2 (mAb 7C7) [24] and polyclonal antibody against VP3 (raised against rVP3 from *E.coli*) were used as primary antibodies. Goat anti-mouse antibody (in 5% skim milk), conjugated with HRP, was used as secondary antibody. Detection was done using Enhanced Chemiluminescence (ECL) Plus western blotting detection reagent (GE Healthcare, UK).

### Transmission electron microscopy

Copper grids were allowed to stand on the purified virus sample for 5 minutes, after which the excess sample on the grid was removed by blotting. Negative staining was performed by placing the sample coated grids on 2% phospho tungstic acid (Sigma) for 2 minutes, blotted, air dried and viewed by JEM-1230 (Jeol) transmission electron microscope.

## Abbreviations

CIM: Convective interaction media; CV: Column volume; DEAE: Diethylaminoethyl cellulose; EV-71: Enterovirus 71; HEPES: 2-[4-(2-hydroxyethyl)piperazin-1-yl]ethanesulfonic acid; HFMD: Hand, foot and mouth disease; mAb: Monoclonal antibody; mM: Millimolar; NaCl: Sodium chloride; PEG: Polyethylene glycol; VP: Viral protein.

## Competing interests

The authors declare that they have no competing interests.

## Authors’ contributions

ARKV carried out the experiments and wrote the manuscript. MS supplied reagents and sample for purification. TKK assisted in transmission electron microscopy and proof-reading. PB supplied reagents and sample for purification. JK conceived and designed the experiment, interpreted the data. All authors read and approved the final manuscript.
